# Clinical, humanistic, and economic burden of haemophilia A in China: findings from a real-world survey

**DOI:** 10.1186/s13023-025-04051-1

**Published:** 2025-10-24

**Authors:** Junchao Feng, Lei Dou, Jingdan Chen, Yunhai Fang, Yan Cheng, Shunping Li

**Affiliations:** 1 Department of Social Medicine and Health Management, School of Public Health, Cheeloo College of Medicine, Shandong University, Jinan, 250012 China; 2 NHC Key Lab of Health Economics and Policy Research, (Shandong University), Jinan, 250012 China; 3 Center for Health Management and Policy Research, Shandong University, (Shandong Provincial Key New Think Tank), Jinan, 250012 China; 4 Center for Health Preference Research, Shandong University, Jinan, Shandong 250012 China; 5 Center for Rare Diseases, Peking Union Medical College Hospital, Chinese Academy of Medical Science and Peking Union Medical College, Beijing, 100730 China; 6 Shandong Blood Center, Shandong Hemophilia Treatment Center, Jinan, 250014 China

**Keywords:** Rare disease, Haemophilia A, Burden, Socio-economic impact, Quality of life, Comprehensive hemophilia care

## Abstract

**Objectives:**

We aimed to describe current treatments for haemophilia A in China, focusing on their associated clinical, humanistic, and economic burdens in a real-world context.

**Methods:**

This was a retrospective cross-sectional study. We investigated the demographics, disease severity, treatment strategies, and clinical outcomes of patients diagnosed with haemophilia A. We also investigated the cost of the treatment of patients with haemophilia A. In the real world, we estimated annual direct medical costs, direct non-medical costs and indirect costs. In addition, we employed the EQ-5D and SF-6D to measure the humanistic burden of patients.

**Results:**

A total of 60 patients were included in the study, comprising 22 children (< 18 years) and 38 adults (≥ 18 years). The mean age of the children and adults was 9.27 and 33.05 years, respectively. Treatment strategies for adults were primarily on-demand. Patients receiving prophylactic treatment experienced fewer bleeds per year compared to those receiving on-demand treatment (mean ABR: adults 42.91 vs. 20.38; children 20.20 vs. 10.10). The mean EQ-5D utility value reported by children and adults were 0.76 (SD 0.24) and 0.51 (SD 0.34), respectively. For adult patients, the SF-6D utility value was 0.38. The mean total annual direct medical costs associated with haemophilia were ¥429,143 (US$58,666) for children and ¥340,238 (US$46,512) for adults, with medication being the primary cost driver.

**Conclusions:**

These data document the enormous burden of haemophilia A that persists in the real world of China. While we emphasize incremental direct healthcare expenditures, we must also consider the long-term clinical and socio-economic benefits of prophylactic treatment.

**Clinical trial number:**

Not applicable.

**Supplementary Information:**

The online version contains supplementary material available at 10.1186/s13023-025-04051-1.

## Background

Haemophilia A is a rare, X-linked haemorrhagic disorder caused by partial or total deficiency of coagulation factor VIII (FVIII) [1]. The prevalence of haemophilia in China ranges from 2.73 to 3.09 per 100,000, with no separate estimate available for haemophilia A [2–5]. Common symptoms in haemophilia A patients include spontaneous bruising, mucosal bleeding, joint bleeding, and epistaxis [6]. Coagulation factor replacement therapy is the most common approach for effectively controlling bleeding in haemophilia patients [7]. It can be categorized into on-demand (administered when bleeding occurs) and prophylactic therapy (regular infusions), with the latter being the recommended treatment strategy [1]. Haemophilia treatment is shifting from frequent infusions of standard half-life factor products toward extended half-life therapies that allow for reduced dosing frequency [8]. In parallel, non-factor therapies have introduced alternative prophylactic approaches that do not rely on traditional clotting factor replacement [9]. While these innovations provide more treatment options and reduce infusion frequency, patients continue to experience a substantial treatment burden [10]. In China, haemophilia has garnered significant attention from the government and society, with a national haemophilia registry established in 1996 [11] and its inclusion in the first national list of rare diseases in 2018 [12]. However, studies indicate that the burden on haemophilia A patients in China remains substantial [5, 13, 14].

The clinical burden of haemophilia A is both persistent and lifelong, with significant detrimental effects. For example, breakthrough bleeding into joints causes disability and pain, while cumulative bleeding often leads to joint deterioration, which may necessitate joint replacement surgery [15]. And the risk of developing inhibitors—antibodies directed against the infused concentrate treatment—is a major adverse event associated with current treatment [16]. Meanwhile, haemophilia is a high-cost disease for both patients and society [5, 17]. The severe symptoms of the disease, complications, adverse effects of treatment, and the significant financial burden impact patients’ quality of life (QoL) [18, 19]. In a previous study, the mean utility value of Chinese haemophilia A patients were significantly lower than those of the general population (0.65 vs. 0.79) [18]. In summary, the burdens faced by patients with haemophilia A are varied, and it is essential to focus on the clinical, economic, and humanistic burdens experienced by these patients in comprehensive assessments.

Previous studies have estimated the clinical, humanistic, and economic burden of haemophilia in Europe and the US using data from CHESS (Cost of Haemophilia in Europe/US: a Socioeconomic Survey) [20–22]. The findings indicate that haemophilia severity is associated with increased economic and humanistic burdens, and that substantial needs among haemophilia patients remain unmet [22]. In China, studies have been conducted to assess the direct medical costs of patients using data from health insurance databases [23] and registry systems [5]. However, current Chinese studies have primarily focused on direct medical costs and have insufficiently considered other types of costs, such as non-medical costs and indirect costs [14]. Additionally, a national survey conducted in 2021 assessed QoL in adult patients but did not include the paediatric patients [24]. Studies conducting comprehensive comparisons between inhibitor and non-inhibitor patients in real-world settings were limited in scope.

We aimed to describe current treatments for haemophilia A in China, focusing on their associated clinical, humanistic, and economic burdens in a real-world context for Chinese patients. Both adults and children, as well as inhibitor and non-inhibitor patients, were included to provide a more comprehensive picture.

## Methods

### Study design and participants

This was a retrospective cross-sectional study. The online survey was conducted between 1 September 2021 and 31 January 2022. We distributed the questionnaire to patients via WeChat (Version 8.0, Tencent, China). After patients became familiar with the questionnaire, investigators conducted follow-up communications online using “Tencent Meeting” (Version 3.0, Tencent, China), which supports screen sharing and real-time video communication. During the communication process, the investigator completed the questionnaire according to the patient’s response. We recruited patients through 5 healthcare institutions (Shandong Hemophilia Treatment Center, Henan Cancer Hospital, Jiangxi Provincial People’s Hospital, The Affiliated Hospital of Xuzhou Medical University, and Shenzhen Children’s Hospital), which are located in the 5 provinces of Shandong, Henan, Jiangsu, Jiangxi, and Guangdong. The provinces are situated in the north, center, and south of China, making them highly representative (Supplementary Figure S1). We classified patients aged ≥ 18 years as adults and those < 18 years as pediatric patients, with the latter’s responses being provided by their primary caregivers.

The eligibility criteria for this study included patients or caregivers who were (1) affected by haemophilia A; (2) receiving treatments for more than 1 year; (3) a primary caregiver who was familiar with the entire treatment process for children; and (4) able to give consent and willing to participate.

On average, the survey lasted approximately 70 min per participant. To express our gratitude for their time and effort, we provided each participant with a letter of thanks and a subsidy (¥100, approximately $14) upon completion of the survey. This study was screened and approved by the Ethics Committee of the Centre for Health Management and Policy Research of Shandong University (No. ECSHCMSDU20200601). As the other five participating centers were only involved in informing patients and no identifiable data were collected, approval from the lead institution was sufficient under local ethical regulations.

### Study outcomes and analyses

The questionnaire included patient characteristics, clinical outcomes, economic costs, and humanistic burdens. We collected data on patient characteristics, including demographic information (province, gender, age, employment status, weight, health insurance) and disease- and treatment-related details (clotting factor level, inhibitors, treatment strategy).

Clinical outcomes included frequency of bleeding events, annualized bleeding rate (ABR), bleed-related hospitalizations, comorbidities, target joints and joint surgery. In this context, “bleeding” was defined as subcutaneous hemorrhage, hematomas, muscular bleeding, joint bleeding, and intracranial hemorrhage—bleeding events that can be perceived by the patient or that require treatment. The target joint is defined as a single joint experiencing 3 or more spontaneous bleeds within a consecutive six-month period [25]. Comorbidities, surgeries, and hospitalizations were reported by the doctor based on a review of the medical records in the hospital’s information system and patient feedback on current treatment. When reports were inconsistent, the investigator verified the information with both the doctor and the patient or caregiver.

We used utility value to represent the humanistic burden based on previous research [21, 26–28]. EQ-5D-5 L and SF-6D were used for adult patients and EQ-5D-Y for children. The Supplementary material Table S1 provides details of the psychometric properties of the different instruments and the value set used.

Economic outcomes encompassed medicine costs, direct medical costs, direct non-medical costs, out-of-pocket direct medical costs, and indirect costs (Supplementary Table S2). Medicine costs were defined as expenditures related to the purchase of prophylactic and on-demand treatments, as well as immune tolerance induction therapies (e.g., prothrombin complex concentrates, plasma-derived factor VIII, recombinant FVIIa, recombinant FVIII, and non-factor products). Direct medical costs included medicine costs, consultations with haemophilia specialists and the multidisciplinary team, testing and hospital stays. Non-medical costs were for the use of assistive devices and home modifications, professional care, as well as travel and accommodation expenses. Out-of-pocket direct medical costs refer to expenses paid by individuals directly for healthcare services, without reimbursement from insurance or other third-party payers. Indirect costs were defined as those related to absenteeism, presenteeism, early retirement, or forced unemployment due to the disease burden, valued using the human capital approach [29, 30]. When calculating costs, patients were asked to provide accurate information, including hospital expense invoices where necessary.

Descriptive statistics were used to summarize the results. For continuous variables, results were expressed as mean and standard deviation or median and range; for categorical variables, results were expressed as number of patients and proportion. All analyses were performed using Excel (Microsoft Office 2019, Microsoft Corporation, USA) and Stata 15 (StataCorp LLC, USA).

## Results

Out of the 70 participants who initially agreed to participate, 60 completed the survey, as shown in Supplementary Figure S2. The 60 participants consisted of 22 children and 38 adults. The characteristics of the participants are summarized in Table 1. The mean ages of adults and children were 33.05 and 9.27 years, respectively. The average weight of adult patients was 68.89 kg. 76.67% of patients had clotting factor levels in the severe group (< 1 IU/dL). Treatment strategies for adults remained predominantly on-demand, while more than half of child patients received prophylactic treatment. A high financial burden was the primary reason for 78.57% (11/14) of patients’ reluctance to receive prophylactic treatment. Furthermore, 25 of the 60 participants had inhibitors (12 children and 13 adults). The time from the first symptom to diagnosis was reported to be within 12 months for all child patients.

**Table 1:** Characteristics of surveyed hemophilia A patients

	All patients (*N* = 60)	Children (*N* = 22)	Adults (*N* = 38)
**Age**,** mean (SD)**	24.33(13.72)	9.27 (3.71)	33.05(8.92)
**Weight**,** kg**,** mean (SD)**	55.5 (23.23)	32.36 (14.61)	68.89(15.38)
**Province**,** N (%)**			
Shandong	27 (45.00)	9 (40.91)	18 (47.37)
Jiangxi	9 (15.00)	4 (18.18)	5 (13.16)
Jiangsu	7 (11.67)	0 (0)	7 (18.42)
Henan	9 (15.00)	1 (4.55)	8 (21.05)
Guangdong	8 (13.33)	8 (36.36)	0 (0)
**Residency type**,** N (%)**			
Urban	27 (45.00)	10 (45.45)	17 (44.74)
Rural	33 (55.00)	12 (54.55)	21 (55.26)
**Dropout of school**,** N (%)**	19 (31.67)	6 (27.27)	13 (34.21)
**Educational level**,** N (%)**			
Primary	-	-	9 (23.68)
Junior high	-	-	9 (23.68)
Senior high	-	-	12 (31.58)
University and above	-	-	8 (21.05)
**Health insurance**,** N (%)**			
UEBMI	8 (13.33)	0(0)	8 (21.05)
RBMI	52 (86.67)	22 (100.00)	30 (78.95)
**Clotting factor level**,** N (%)**			
< 1 IU/dL (severe)	46 (76.67)	18 (81.82)	28 (73.68)
1 to < 5 IU/dL (moderate)	10(16.67)	4 (18.18)	6 (15.79)
5 to < 40 IU/dL (mild)	4 (6.67)	0 (0)	4 (10.53)
**Treatment strategy**,** N (%)**			
On-demand	36 (60.00)	9 (40.91)	27 (71.05)
Standard-dose prophylaxis	16 (26.67)	10 (45.45)	6 (15.79)
Low-dose prophylaxis	8 (13.33)	3 (13.64)	5 (13.16)
**Inhibitor**,** past 12 months**,** N (%)**	25(41.67)	12 (54.55)	13 (34.21)

“-” indicates not applicable; UEBMI: urban employee basic medical insurance; RBMI: resident basic medical insurance

### Clinical outcomes

Table 2 presents the clinical outcomes of patients with haemophilia. A higher proportion of child patients received prophylactic treatment and experienced fewer bleeding episodes compared to adult patients. Patients on prophylactic treatment had fewer annual bleeds compared to those on on-demand therapy (mean ABR: adults 42.91 vs. 20.38; children 20.10 vs. 10.72). Synovitis and haemophilic arthropathy were the most common comorbidities. Aside from synovitis, comorbidities were more severe in adults than in children. Approximately 40% of adult patients underwent joint surgery. Children on prophylactic treatment had a high number of outpatient visits. We observed that children receiving prophylactic treatment had fewer bleed-related hospital days than those treated on-demand, a difference not observed in adults. In the same age group, patients with inhibitors had higher ABR and more hospital bed days than those without inhibitors (Supplementary Table S3). The knee is the joint most commonly affected by hemorrhage (Supplementary Figure S3).

**Table 2:** Clinical outcomes

Clinical outcomes	All patients (*N* = 60)	Children (*N* = 22)			Adults (*N* = 38)		
		Total	Prophylaxis (*N* = 13)	On-demand (*N* = 9)	Total	Prophylaxis (*N* = 11)	On-demand (*N* = 27)
**ABR**,** mean (SD)**	28.54 (34.94)	14.97 (12.00)	10.72 (8.10)	21.10 (14.42)	36.39 (41.14)	20.38 (18.47)	42.91 (46.10)
**ABR**,** Median (Range)**	15.15	12.12	10.10 (1.01–26.26)	20.20 (4.04–46.46)	21.71	15.15 (3.03–60.61)	23.23 (3.03- 151.52)
**Number of bleeds per year**,** N (%)**							
< 5	7 (11.48)	4 (18.18)	3 (23.08)	1 (11.11)	3 (7.89)	2 (18.18)	1 (3.70)
5–20	26 (42.62)	11 (50.00)	8 (61.54)	5 (55.56)	15 (39.47)	5 (45.45)	11 (40.74)
21–50	18 (29.51)	7 (31.82)	2 (15.38)	3 (33.33)	11 (28.95)	3 (27.27)	7 (25.93)
> 51	10 (16.39)	0 (0.00)	0 (0.00)	0 (0.00)	9 (23.68)	1 (9.09)	8 (29.63)
**Comorbidities**,** N (%)**							
Synovitis	40 (66.67)	14 (63.64)	10(76.92)	4 (44.44)	26(68.42)	9 (81.82)	17 (62.96)
Hemophilia arthropathy	37 (61.67)	7 (31.82)	4 (30.77)	3 (33.33)	30 (78.95)	8 (72.73)	22 (81.48)
Pseudotumor	8 (13.33)	0 (0.00)	0 (0.00)	0 (0.00)	8 (21.05)	2 (18.18)	6 (22.22)
HIV	0 (0.00)	-	-	-	-	-	-
Hepatitis B	1 (1.67)	0 (0.00)	0 (0.00)	0 (0.00)	1(2.63)	0 (0.00)	1 (3.7)
Hepatitis C	3 (5.00)	0 (0.00)	0 (0.00)	0 (0.00)	3 (7.89)	2 (18.18)	1 (3.7)
**History of joint surgery**,** N (%)**	16 (26.67)	1 (4.55)	1 (7.69)	0 (0.00)	15 (39.47)	4 (36.36)	11 (40.74)
**Bleeding-related outpatient visit**,** mean (SD)**	16.56 (26.90)	22.31 (35.96)	22.38 (44.78)	22.22 (19.66)	13.23 (19.71)	13.90 (16.96)	12.96(21.02)
**Bleed-related hospitalisations**,** mean (SD)**	1.5 (2.80)	1.76 (3.46)	2.13 (1.96)	1.54 (4.20)	1.34 (2.35)	0.55 (1.21)	1.71 (2.66)
**Bleed-related hospital days per patient**,** mean (SD)**	17.79 (19.79)	20.4 (24.25)	4.50 (5.45)	31.00 (26.53)	16.70 (18.10)	12.40 (17.64)	17.84 (18.51)

“-” indicates not applicable

### Humanistic outcomes

As shown in Table 3, children and adults reported mean EQ-5D utility value of 0.76 (SD 0.24) and 0.51 (SD 0.34), respectively. The utility value for adult patients, as measured by the SF-6D (0.38), was lower than that measured by the EQ-5D. Nearly half of the surveyed patients (46.67%) reported feelings of loneliness in their daily lives, while approximately 80% reported good family relationships. The majority of patients (> 80%) reported that haemophilia compromised their daily lives. The EQ-5D utility value for children and adult inhibitor patients were 0.66 and 0.41, respectively, with differences of 0.21 and 0.15 compared to non-inhibitor patients (Supplementary Table S3).

**Table 3:** Humanistic outcomes

Humanistic outcome	All patients (*N* = 60)	Children (*N* = 22)			Adults (*N* = 38)		
		Total	Prophylaxis (*N* = 13)	On-demand (*N* = 9)	Total	Prophylaxis (*N* = 11)	On-demand (*N* = 27)
**EQ-5D utility value**,** mean (SD)**	-	0.76 (0.24)	0.80 (0.20)	0.69 (0.29)	0.51(0.34)	0.53 (0.18)	0.50(0.39)
**SF-6D utility value**,** mean (SD)**	-	-	-	-	0.38 (0.28)	0.38 (0.31)	0.37 (0.28)
**Sense of loneliness**,** N (%)**							
Yes	28 (46.67)	7 (31.82)	4 (30.77)	3 (33.33)	21 (55.26)	7 (63.64)	14(51.85)
No	32(53.33)	15 (68.18)	9 (69.23)	6 (66.67)	17 (44.74)	4 (36.36)	13 (48.15)
**Family Relationships**,** N (%)**							
Good	52 (86.67)	21 (95.45)	13 (100.00)	8 (88.89)	31 (81.58)	7 (63.64)	24 (88.89)
Poor	8 (13.33)	1 (4.55)	0 (0.00)	1 (11.11)	7 (18.42)	4 (36.36)	3 (11.11)
**Daily life compromised by hemophilia**							
Yes	52 (86.67)	17 (77.27)	8 (61.54)	9 (100.00)	35 (92.11)	9 (81.82)	26 (96.30)
No	8 (13.33)	5 (22.73)	5 (38.46)	0 (0.00)	3 (7.89)	2 (18.18)	1 (3.70)
**Worried about the future life**,** N (%)**							
Yes	35 (58.33)	11 (50.00)	4 (30.77)	7 (77.78)	24 (63.16)	8 (72.73)	16 (59.26)
No	25(41.67)	11 (50.00)	9 (69.23)	2 (22.22)	14 (36.84)	3 (27.27)	11 (40.74)

“-” indicates not applicable

### Cost outcomes

**Fig. 1:**
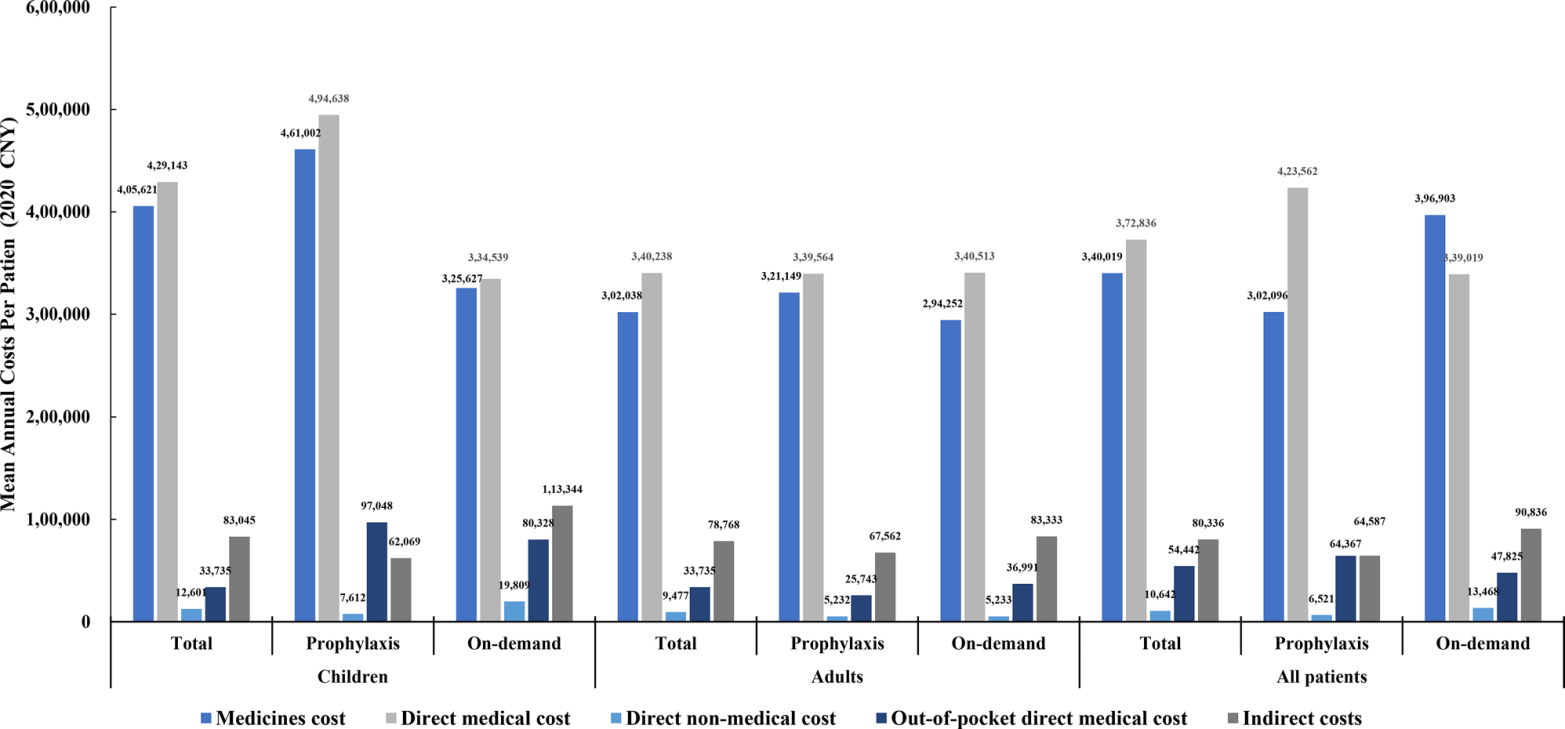
Comparison of mean cost in different categories

**Fig. 2:**
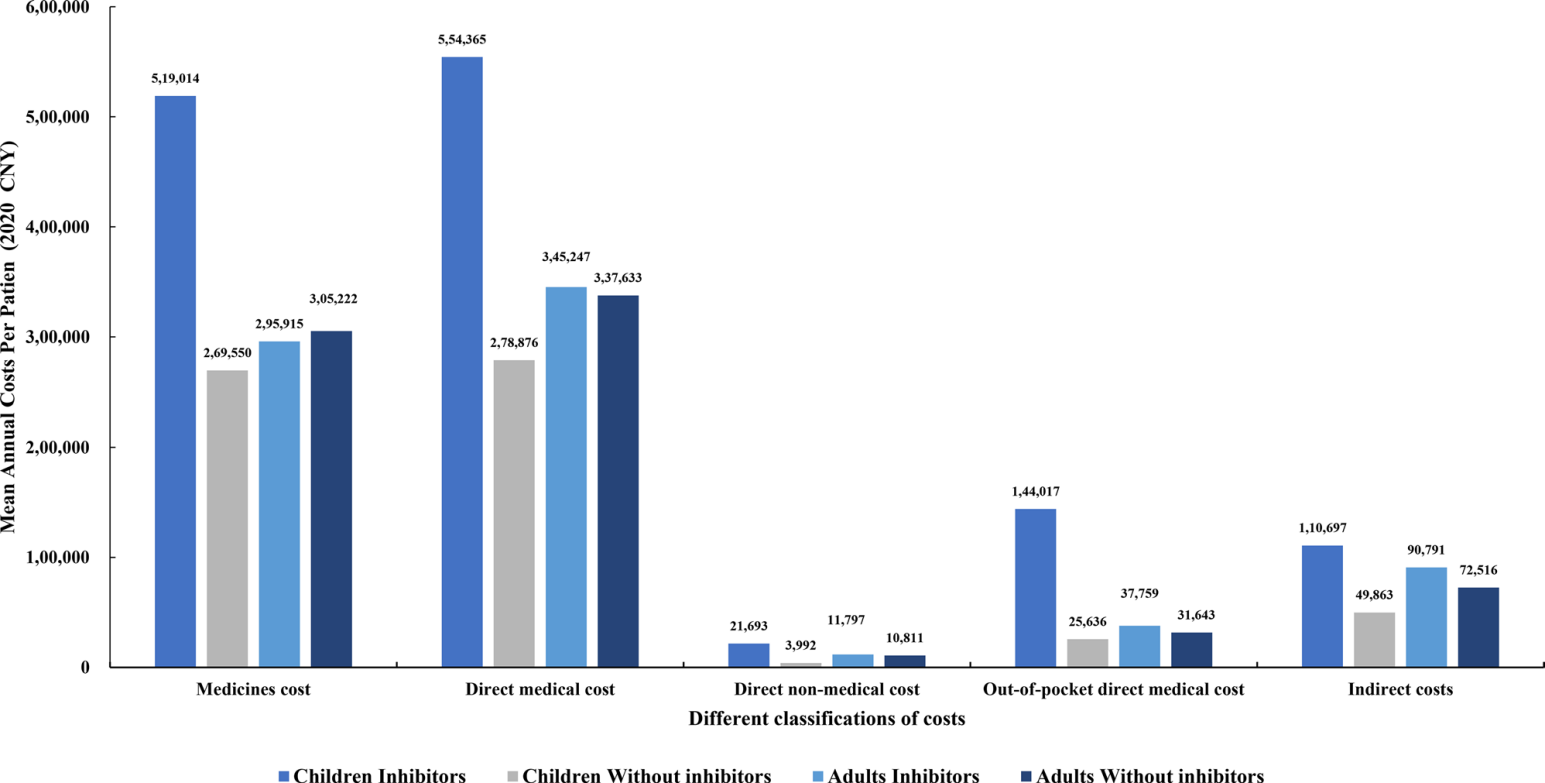
Comparison of different categories of mean cost in inhibitor and non-inhibitor patients

The mean annual total direct medical costs associated with haemophilia for child and adult patients were ¥429,143 ($58,666) and ¥340,238 ($46,512), respectively, with the costs of medicines accounting for 86–97% (Fig. 1). The mean annual direct non-medical costs for children and adults were approximately equivalent, primarily driven by professional care and travel expenses. Direct non-medical costs were lower for both children and adults receiving prophylactic treatment compared to those on on-demand treatment. Haemophilia-related job loss and early retirement incurred annual indirect costs ranging from ¥60,000 to ¥120,000 ($8,202 − 16,404), with the highest costs observed in the on-demand treatment group of children. The average annual out-of-pocket costs for adults were ¥34,725 ($4,747), which was slightly higher than the ¥33,735 ($4,611) for children. Across all cost categories, child patients with inhibitors incurred the highest expenses, reaching ¥554,365 ($75,784) in direct medical costs. The cost differences between inhibitor and non-inhibitor child patients were significant across all categories. For instance, the average annual direct medical costs for child patients with inhibitors were nearly twice as high as for those without inhibitors (¥554,365/$75,784 vs. ¥278,876/$38,123). However, cost differences between adult patients with and without inhibitors were not significant (Fig. 2).

## Discussion

To the best of our knowledge, this is the first comprehensive study to assess both the disease and economic burden of haemophilia patients in China within a real-world context. This real-world analysis has demonstrated that haemophilia A has a persistent negative impact on Chinese patients. Despite concerted efforts by the Chinese government to improve treatment accessibility and affordability, our analysis reveals a persistent and extensive clinical, humanistic, and economic burden of haemophilia A, with substantial costs incurred by patients, payers, and society. In addition to the high treatment costs, personal and social burdens were also evident, along with diminished QoL for patients. These findings underscore the substantial unmet need regarding the burden of haemophilia A in both adult and child patients in China.

We found that approximately 29% of adult haemophilia A patients in China, and 59% of children, received prophylactic treatment (Table 1). According to the 2018 World Federation of Haemophilia Annual Global Report, the proportion of Chinese minors with haemophilia receiving prophylactic treatment is about 15%, and about 7% for adults [31]. It is evident that the proportion of patients receiving prophylactic treatment is increasing in China; however, it remains low compared with that in developed countries and regions. Approximately 75% of patients with haemophilia in the United States receive prophylactic treatment, and more than 80% in the United Kingdom [32]. Similar to findings from other studies, the financial burden remains the primary barrier preventing Chinese haemophilia patients from accessing prophylactic treatment [13, 33, 34]. In China, the benefits vary significantly between different cities, particularly in aspects such as the reimbursement rate (ranging from 40% to 90%), the insurance scheme’s upper limits, and whether patients can claim reimbursements from both basic medical insurance and critical illness insurance simultaneously [35]. These variations significantly impact patients’ out-of-pocket medical costs, consequently influencing their willingness to accept and maintain prophylactic treatment [34]. These huge gaps suggest that China needs to continue to improve the accessibility of haemophilia A medicine and the affordability of prophylactic treatment.

As a portion of patients undergo on-demand treatment, individuals with haemophilia A continue to experience breakthrough bleeding, potentially leading to complications such as synovitis, haemophilic arthropathy, and pseudotumours. The ABR observed in our study was higher than that reported in previous Chinese studies for patients receiving prophylactic treatment (ABR: 8.4 [36]; 2.0 [37]), but lower than that for those undergoing on-demand treatment (ABR: 61.3 [37]). The primary comorbidities among patients were synovitis and haemophilic arthritis associated with bleeding, while complications from viral infections (e.g., HIV, Hepatitis B, and Hepatitis C) were less common. Children exhibit lower rates of all types of comorbidities compared to adults, which is associated with the increased use of prophylactic treatment among the child patients. This is also connected to China’s health insurance policy, which permits reimbursement for prophylactic treatment for children with haemophilia, while adult patients are restricted to receiving medication solely in the event of bleeding (on-demand treatment) [38, 39].

We observe a corresponding degree of humanistic burden, with an average utility value of 0.51 for EQ-5D-5L in adult patients, which aligns with values reported in recent empirical studies in China [24, 40]. Similar to a previous study [41, 42], adult patients exhibited lower health utility value on the SF-6D compared to EQ-5D-5L, which may be attributed to the higher sensitivity of the SF-6D [43]. Children receiving prophylactic treatment in greater proportions exhibited higher EQ-5D-Y health utility value (0.76) than their adult counterparts. Almost half of the patients frequently reported feelings of loneliness, low overall life satisfaction, and concerns about their future. These results highlight the need to prioritize patients’ mental health and social integration.

Haemophilia is a costly yet treatable rare disease that is covered by basic medical insurance reimbursement in China. However, out-of-pocket costs for patients remain substantial due to restrictions on prophylactic treatment for adults and the insurance scheme’s upper limits. Another survey indicates that the mean annual treatment costs for Chinese patients with haemophilia are ¥243,000 ($33,193), with 80% attributed to medication expenses [38]. The elevated patient costs observed in our study compared to previous surveys may stem from a selection bias. Since we recruited patients through hospitals, those included were actively engaged in their treatment. In the real world, we have also observed that better clinical outcomes can reduce absenteeism and unemployment among patients or their caregivers, which in turn lowers indirect costs. Therefore, we advocate for universal access to prophylactic treatment for both children and adults, as well as immune-inducing treatment for patients with inhibitors. While such initiatives may initially increase direct healthcare expenditures, they offer long-term clinical and economic benefits.

In the children group, patients with inhibitors exhibited a significantly higher clinical burden compared to those without inhibitors, as demonstrated by a nearly threefold increase in total mean annual bleeding rate (ABR), number of hospitalizations, and days spent in the hospital, consistent with previous studies [44, 45]. Meanwhile, direct medical costs were highest in the group of children with inhibitors. Child patients with inhibitors also exhibited lower EQ-5D-Y value compared to the non-inhibitor group. In contrast, direct medical costs did not differ significantly between adult patients with inhibitors and those without, indicating that adult patients with inhibitors were not receiving standardized care. Our results revealed that adult patients with inhibitors had lower ABR, fewer bleeding-related outpatient visits, and fewer hospital days than patients without inhibitors. We believe that this does not indicate better clinical outcomes for patients with inhibitors, which may be related to recall bias when adult patients with inhibitors self-report bleeding. This is confirmed by the humanistic results, which indicate that adult patients with inhibitors had significantly lower utility value (EQ-5D-5L and SF-6D) compared to those without inhibitors. Future studies are needed to investigate the differences in clinical outcomes between patients with inhibitors and those without inhibitors in China, as well as the factors influencing these results.

Based on our findings, we propose that support for haemophilia A patients in China should focus on three key areas. First, basic medical insurance should eliminate restrictions on prophylactic treatment for adults, allowing them to access prophylactic treatment with lower out-of-pocket costs, thereby reducing bleeding episodes and associated complications. Second, special attention should be given to patients with inhibitors, who face a greater burden; support could include access to immune tolerance induction therapy or non-factor therapies. Finally, multidisciplinary support programs should be established within the existing China National Haemophilia Cooperative Group, offering training in infusion techniques, rehabilitation, mental health education, and social integration, to enhance patients’ QoL.

## Strengths and limitations

In this study, we recruited patients from 5 provinces in China, ensuring a strong representation by considering both the patients’ ages and their inhibitor status. Furthermore, we provided a comprehensive survey and presentation of the clinical outcomes, humanistic burden, and economic costs associated with the patients. We believe that this detailed information on patients with haemophilia will be valuable for other researchers and inform future policy formulation. More importantly, we conducted a real-world survey that better captures the actual circumstances of patients compared to data from registration databases. However, this study has several limitations. First, although the patients with haemophilia recruited were from across the country, the total number of participants was relatively small. Second, like all retrospective studies, our research is susceptible to selection bias and recall bias. Since patient purchases were not limited to a single surveyed hospital, accurately recalling the amount of FVIII consumed proved challenging.

## Conclusion

The findings of our real-world analyses demonstrate that haemophilia A imposes a significant clinical and humanistic burden on patients, alongside a substantial economic burden. Our analyses provide a comprehensive assessment of the current real-world treatment outcomes for children and adults, including both inhibitor and non-inhibitor patients in China, highlighting that unmet needs persist for these patients, their caregivers, and society.

## Supplementary Information

Below is the link to the electronic supplementary material.

Supplementary Material 1

## Data Availability

Data are available from the authors upon reasonable request.

## Abbreviations

EQ-5D-5L: EuroQol 5-Dimensions 5-Levels
SF-6D: Six-dimensional health state classification system
EQ-5D-Y: EuroQol 5-Dimensions for Youth
QoL: Quality of life
ABR: Annualized Bleeding Rate
