# Supplementary material for: Clinical, humanistic, and economic burden of haemophilia A in China: findings from a real-world survey
Source: Orphanet J Rare Dis. 2025 Oct 24;20:538. doi: 10.1186/s13023-025-04051-1 (PMC12551187; doi:10.1186/s13023-025-04051-1)
Supplement: Supplementary file 1 — Supplementary Material 1 [file 13023_2025_4051_MOESM1_ESM.docx]

**Supplementary Information**


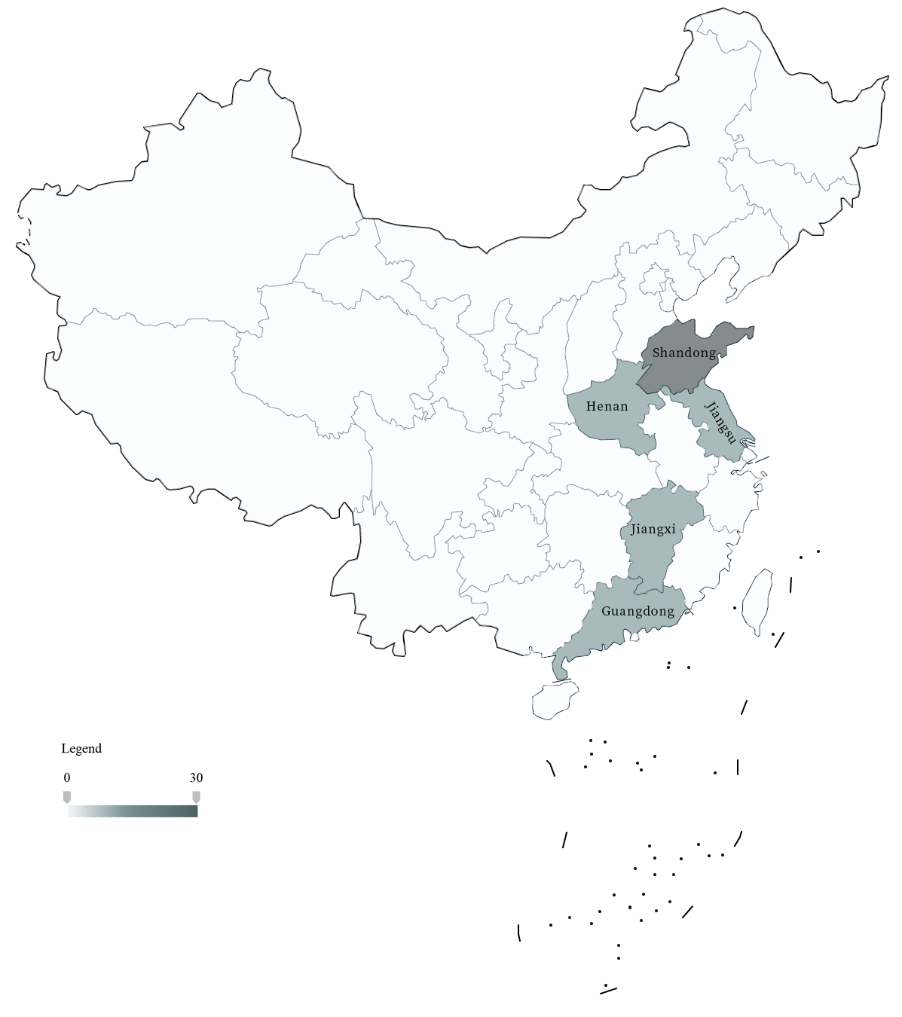


**Figure S1 Map of the distribution of surveyed patients**ꭝ

ꭝThe map was sourced from the China Standard Map Service website (http://bzdt.ch.mnr.gov.cn/index.html)

**Table S1 Instruments used by different age groups**

|  | **EQ-5D** | | **SF-6D** ^1^ |
| --- | --- | --- | --- |
|  | **ED-5D-5L** ^2^ | **ED-5D-Y** ^3^ |  |
| **Domains** | 5  (mobility, self-care, usual activities, pain/discomfort and anxiety/depression) | 5  (walking about, looking after myself, doing usual activities, having pain/discomfort, and feeling worried/ sad/ unhappy) | 6  (physical functioning, role limitation, social functioning, bodily pain, mental health and vitality) |
| **Levels** | 5  (no problems, slight problems, moderate problems, severe problems and extreme problems) | 3  (no problems, some problems, and a lot of problems) | 5/6  (none of the time, a little of the time, some of the time, most of the time, all of the time) |
| **Valuation technique** | TTO | TTO | TTO |
| **Possible health states** | 3125 | 243 | 18750 |
| **Value set** | Chinese value set ^4^ | Chinese value set ^5^ | Chinese value set ^6^ |
| **Boundaries** | -0.391 to 1 | -0.088 to 0.924 | -0.277 to 1 |
| **Target patient group** | Adult group | Children group | Adult group |

TTO: time trade-off

**Table S2 Cost components**

| **Outcome** | **Measured element** | **Description and definition** |
| --- | --- | --- |
| Medicines costs | Prothrombin complex concentrates | Costs associated with medications for haemophilia prophylaxis or on-demand treatment. |
|  | Plasma-derived factor VIII |  |
|  | Recombinant FVIIa |  |
|  | Recombinant FVIII |  |
|  | Non-factor products |  |
| Direct medical costs | Medicines | Ibid |
|  | Consultant visits | Costs associated with consultations at medical institutions resulting from haemophilia and its related complications. |
|  | Hospitalisations | Hospitalization costs resulting from haemophilia and its associated complications. |
|  | Tests and examinations | The costs associated with diagnostic tests for haemophilia and its complications, including blood tests, joint ultrasound examinations and other tests. |
| Out-of-pocket direct medical cost^✝^ | Individuals pay directly from their own finances for direct medical costs, without reimbursement from insurance or other third-party payers. | |
| Non-medical costs | Assistive devices and home alterations | For the purchase of assistive devices or the costs associated with home modifications, such as walking aids and home adjustments. |
|  | Professional care | Costs associated with professional care resulting from haemophilia and its complications. |
|  | Travelling and accommodation expenses | Travel and accommodation costs incurred for consultant visits, medication purchases, and hospitalizations. |
| Indirect costs | Work productivity impact | Costs associated with absenteeism and early retirement resulting from haemophilia and its complications. |
|  | Caregiver burden | Costs associated with caregiver absenteeism and early retirements resulting from the care of haemophilia patients. |

Note: All data are derived from real-world patient/caregiver reports, and to ensure the authenticity of the information, patients provide invoices, receipts, or payment documentation with their personal information anonymized.

^✝^Direct medical costs, including medication expenses, constitute the primary cost category, and are the main source of patients' out-of-pocket expenses. Based on previous studies, we use the term 'out-of-pocket direct medical cost^7^.

**Table S3 Comparison of major clinical and humanistic outcomes in inhibitor and non-inhibitor patients**

|  |  | **Children (N=22)** | |  | **Adults (N=38)** | |
| --- | --- | --- | --- | --- | --- | --- |
| **Main outcome** |  | **Inhibitors**  **(N= 12)** | **Without inhibitors(N=10)** |  | **Inhibitors**  **(N=13)** | **Without inhibitors (N=25)** |
| **ABR, mean (SD)** |  | 21.21 (12.69) | 7.47 (5.00) |  | 32.17(47.57) | 38.58(38.25) |
| **ABR, Median (Range)** |  | 20.20 (4.04-46.46) | 7.58 (1.01- 15.15) |  | 10.10(3.03-151.52) | 23.23 (3.03-121.21) |
| **Number of bleeds per year, N (%)** |  |  |  |  |  |  |
| <5 |  | 1 (8.33) | 3 (30.00) |  | 2 (15.38) | 1(4.00) |
| 5-20 |  | 6 (50.00) | 7 (70.00) |  | 6 (46.15) | 10 (40.00) |
| 21-50 |  | 5 (41.67) | 0 (0.00) |  | 3 (23.08) | 7 (28.00) |
| >51 |  | 0 (0.00) | 0 (0.00) |  | 2 (15.38) | 7 (28.00) |
| **History of joint surgery, N (%)** |  | 0 (0.00) | 1 (10.00) |  | 3 (23.08) | 12 (48.00) |
| **Bleeding-related outpatient visit, mean (SD)** |  | 19.25 (18.33) | 26.00 (50.79) |  | 9.77 (13.80) | 15.04 (22.22) |
| **Bleed-related hospitalisations, mean (SD)** |  | 2.75(4.25) | 0.4 (1.26) |  | 1.38 (1.12) | 1.16 (2.72) |
| **Bleed-related hospital days per patient, mean (SD)** |  | 16.00 (23.82) | 1.2 (3.79) |  | 8.69 (7.52) | 11.52 (19.63) |
| **EQ-5D utility value, mean (SD)** |  | 0.66 (0.29) | 0.87 (0.08) |  | 0.41 (0.35) | 0.56 (0.33) |
| **SF-6D utility value, mean (SD)** |  | - | - |  | 0.23 (0.27) | 0.45 (0.26) |

"-" indicates not applicable

**N=70**

30 from Shandong, 10 each from Jiangsu, Henan, Guangdong and Jiangxi

**N=61**

Questionnaire too long (N=4);

Concerns about child's privacy being revealed (N=3);

No clear memory of details of spending (N=2)

Missing data in the questionnaires (N=1)

**N=60**

**Figure S2 Flow diagram on enrolment procedures for haemophilia A patients**

**Figure S3 Target joints most affected by patient reports**

**Reference**

1. Wu J, Xie S, He X, et al. The Simplified Chinese version of SF-6Dv2: translation, cross-cultural adaptation and preliminary psychometric testing.*Qual Life Res*. 2020;29(5):1385-91.

2. Devlin NJ, Brooks R. EQ-5D and the EuroQol Group: Past, Present and Future.*Appl Health Econ Health Policy*. 2017;15(2):127-37.

3. Golicki D, Młyńczak K. Measurement Properties of the EQ-5D-Y: A Systematic Review.*Value Health*. June 2022;S1098-3015(22)02001-0.

4. Yang Z, Busschbach J, Liu G, et al. EQ-5D-5L norms for the urban Chinese population in China.*Health Qual Life Outcomes*. 2018;16(1):210.

5. Yang Z, Jiang J, Wang P, et al. Estimating an EQ-5D-Y-3L Value Set for China.*Pharmacoeconomics*. 2022;40(Suppl 2):147-55.

6. Wu J, Xie S, He X, et al. Valuation of SF-6Dv2 Health States in China Using Time Trade-off and Discrete-Choice Experiment with a Duration Dimension.*Pharmacoeconomics*. 2021;39(5):521-35.

7. Li Z, Wu J, Zhao Y, et al. Influence of medical insurance schemes and charity assistance projects on regular prophylaxis treatment of the boys with severe haemophilia A in China.*Haemophilia*. 2018;24(1):126-33.
